# Optogenetic control of contractile function in skeletal muscle

**DOI:** 10.1038/ncomms8153

**Published:** 2015-06-02

**Authors:** Tobias Bruegmann, Tobias van Bremen, Christoph C. Vogt, Thorsten Send, Bernd K. Fleischmann, Philipp Sasse

**Affiliations:** 1 Institute of Physiology I, University of Bonn, Life and Brain Center, Sigmund-Freud-Strasse 25, 53127 Bonn, Germany; 2 Research Training Group 1873, University of Bonn, 53127 Bonn, Germany; 3 Department of Otorhinolaryngology/Head and Neck Surgery, University Hospital of Bonn, Sigmund-Freud-Strasse 25, 53127 Bonn, Germany

## Abstract

Optogenetic stimulation allows activation of cells with high spatial and temporal precision. Here we show direct optogenetic stimulation of skeletal muscle from transgenic mice expressing the light-sensitive channel Channelrhodopsin-2 (ChR2). Largest tetanic contractions are observed with 5-ms light pulses at 30 Hz, resulting in 84% of the maximal force induced by electrical stimulation. We demonstrate the utility of this approach by selectively stimulating with a light guide individual intralaryngeal muscles in explanted larynges from ChR2-transgenic mice, which enables selective opening and closing of the vocal cords. Furthermore, systemic injection of adeno-associated virus into wild-type mice provides sufficient ChR2 expression for optogenetic opening of the vocal cords. Thus, direct optogenetic stimulation of skeletal muscle generates large force and provides the distinct advantage of localized and cell-type-specific activation. This technology could be useful for therapeutic purposes, such as restoring the mobility of the vocal cords in patients suffering from laryngeal paralysis.

## Abstract (web summary)

Nerve damage can lead to skeletal muscle paralysis. The authors show that localized photostimulation of skeletal muscle expressing the light-sensitive channel Channelrhodopsin-2 generates contraction in the absence of neural impulses and prove that this strategy can be used for optogenetic opening of explanted murine vocal cords.

## Graphical abstract

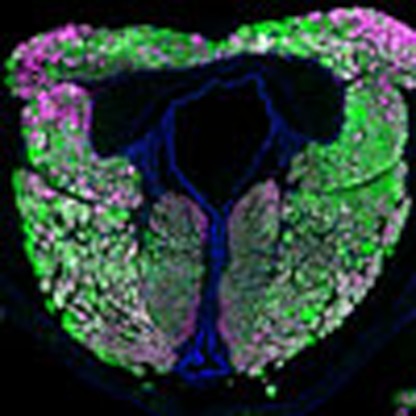

In contrast to electrical stimulation, optogenetic methods allow cell-type-specific stimulation with high spatial and temporal precision of excitable cells by expressing light-sensitive proteins such as the light-gated nonselective cation channel Channelrhodopsin-2 (ChR2)^1^,^2^,^3^. This technology has been used to evoke contractions in innervated skeletal muscle by indirect stimulation of either the secondary motor cortex^3^ or of peripheral motor neurons^4^,^5^,^6^, whereas direct optogenetic stimulation of skeletal muscle cells was so far shown only in nematodes^7^ and in immortalized myotubes *in vitro*^8^. These as well as earlier studies in heart muscle^9^,^10^ do not allow to predict the feasibility and efficacy of direct optogenetic stimulation of intact mammalian skeletal muscle. This is because maximal force generation in intact skeletal muscle depends on the recruitment of electrically isolated muscle fibres, which is potentially challenging when using direct optogenetic stimulation of skeletal muscle because of the high concentration of light-absorbing myoglobin. Furthermore, maximal and sustained force can only be generated by Ca^2+^ accumulation during tetanic high-frequency stimulation, which is in contrast to the non-tetanic heart.

Because of these specific features of skeletal muscle physiology, we explore herein the biophysical basics of direct optogenetic stimulation of intact mammalian skeletal muscle. Muscle-specific expression of ChR2 would allow the spatially controlled and pain-free stimulation of skeletal muscle, and therefore we test also the applicability of optogenetic stimulation of individual intralaryngeal muscles to illustrate as a proof-of-concept the functional recovery of paralysed larynges.

## Results

### Direct optogenetic stimulation of isolated muscle fibres

**Figure 1:**
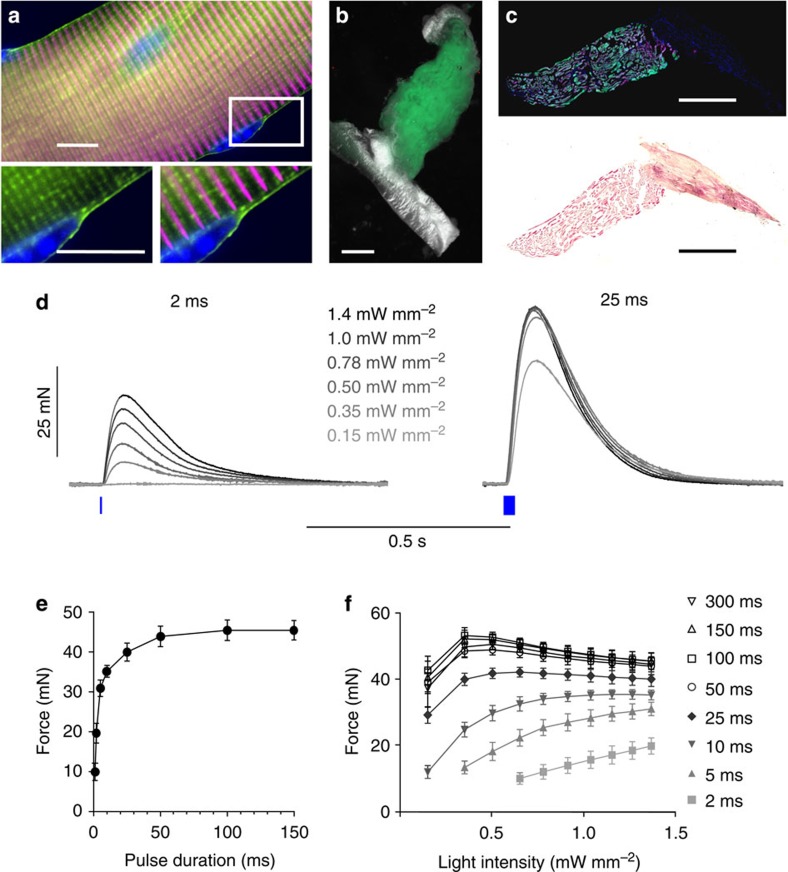
Functional expression of ChR2 in skeletal muscle. (**a**–**c**) Expression of ChR2-EYFP in skeletal muscles. (**a**) Single fibres isolated from flexor digitorum brevis muscles showed bright ChR2-EYFP (green) signals with localization at the cell membrane including the t-tubulus system that surrounds the α-actinin (magenta) containing z-discs (enlargements in lower panels). (**b**,**c**) Bright ChR2-EYFP (green) signals were found in explanted soleus muscles (**b**) and were restricted to α-actinin (magenta) positive skeletal muscle cells (**c**, top). No expression was seen in the tendon identified with haematoxylin and eosin staining (**c**, bottom). (**d**–**f**) Light-induced single twitches in soleus muscles. (**d**) Representative examples of single twitches induced by 2- and 25-ms-long light pulses with increasing light intensities. (**e**) Relationship of pulse duration and maximal twitch force at high light intensity (1.4 mW mm^−2^, *n*=5). (**f**) Overall comparison of the maximum twitch force induced by single light pulses of different durations and intensities (*n*=5). Error bars, s.e.m., nuclear staining in blue, scale bars, 10 μm (**a**), 1 mm (**b**,**c**).

We used transgenic mice expressing the ChR2(H134R) mutant in fusion with enhanced yellow fluorescent protein (EYFP) under the control of the chicken-β-actin promotor^10^, which is strongly active in muscle cells^11^, and found bright EYFP signals in single flexor digitorum brevis (FDB) fibres located within the plasma membrane and in the t-tubulus system (Fig. 1a). Illumination of isolated single FDB fibres with short light flashes (470 nm, 1 ms, 8 mW mm^−2^) induced contractions (Supplementary Movie 1) proving the direct activation of muscle because isolated single FDB fibres do not contain neuromuscular synapses.

### Light-induced isometric force in intact soleus muscles

To quantify the potency of optogenetic stimulation in intact skeletal muscles, isometric force was measured in explanted soleus muscles, which showed EYFP expression (Fig. 1b) in α-actinin-positive muscle fibres (Fig. 1c). Stimulation with light pulses as short as 2 ms and as low as 0.35 mW mm^−2^ induced contractions and force gradually increased with higher light intensities (Fig. 1d left). When using 25-ms-long pulses, force generation saturated at low light intensities (0.5 mW mm^−2^; Fig. 1d right). Importantly, with high light intensity (1.4 mW mm^−2^) force could be increased by prolongation of light pulses from 1 to 50 ms, whereas longer light pulses did not further increase force generation (Fig. 1e). Comparison of all light intensities and pulse durations highlighted their interdependence, and longer light pulses led to a saturation of force at lower light intensities (Fig. 1f). Overall, the maximal force of light-induced single twitches was 53.2±2.4 mN (*n*=5) using 100-ms-long light pulses at 0.35 mW mm^−2^.

**Figure 2:**
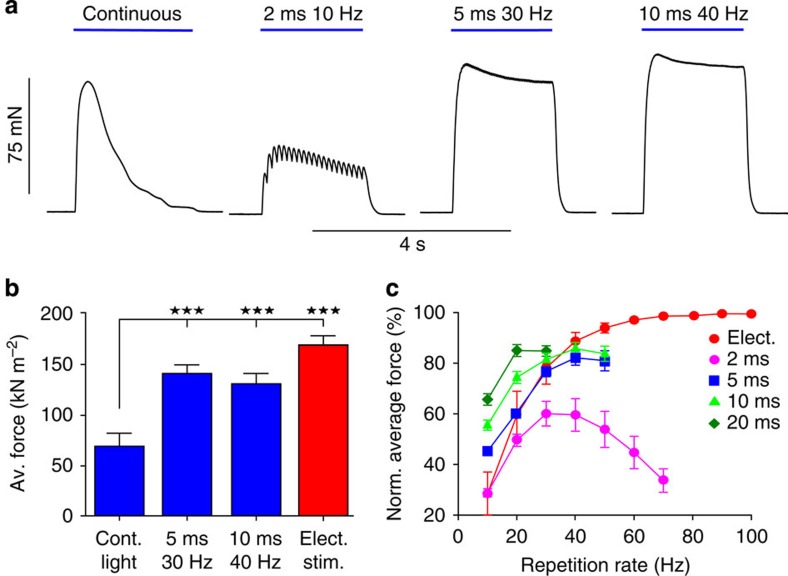
Optogenetic generation of tetanic contractions. (**a**) Representative examples of sustained contractions generated by various 2-s-long illumination patterns. (**b**) Quantification of average force during optical stimulation in comparison with electrical stimulation (100 Hz, 20 V, 1 ms, biphasic) (*P*<0.0001, repeated Dunnett ANOVA test with continuous light stimulation as control, *n*=6). (**c**) Relationship between repetition rate and average force normalized to maximal electrical stimulation for different light pulse durations (*n*=4). Error bars, s.e.m. Av., average; cont., continuous; elect., electrical; norm., normalized; stim., stimulation.

**Figure 3:**
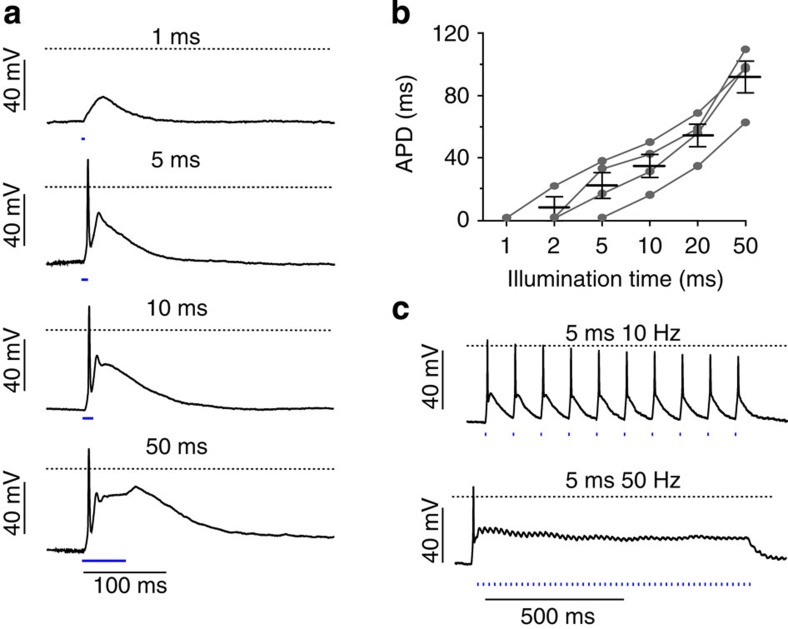
Light-induced action potentials. (**a**) Light-induced depolarizations and action potentials using 1-, 5-, 10- and 50-ms-long light pulses (1.4 mW mm^−2^). (**b**) Relationship between the duration of light pulses and action potential durations (APD, *n*=4). (**c**) Membrane potential change during tetanic stimulation (rate of 10 or 50 Hz) using 5-ms-long light pulses (1.4 mW mm^−2^). Error bars, s.e.m.

In order to obtain maximal force in skeletal muscle, sustained contractions are required. Interestingly, continuous illumination resulted in non-sustained force generation with an initial peak followed by a decline to basal levels (Fig. 2a) highlighting the importance of pulsed stimulation. To determine the most effective pulsed illumination, we tested a broad range of repetition rates (10–70 Hz) and pulse durations (2–20 ms) and analysed the average force during these stimulations. Using low repetition rates (10 Hz), soleus muscles showed incomplete tetanic contractions with short relaxations between the light pulses; however, repetition rates above 30 Hz led to complete and uniform tetanic contractions (Fig. 2a). Overall optical stimulation with 5 ms at 30 Hz generated the highest average force with 94.3±5.6 mN and the average force per cross-sectional area amounts to 140.1±8.4 kN m^−2^ (*n*=6; Fig. 2b). This value was close (84.0±4.7%) to the maximal force induced by the most effective electrical stimulation. To analyse the force–frequency relationship of optical compared with electrical stimulation, we normalized for each muscle the respective force on optical stimulation with the maximal force induced by electrical stimulation (Fig. 2c). At low repetition rates (<30 Hz), pulsed illumination was superior to electrical stimulation in generating force; however, the efficacy of optical stimulation decreased at repetition rates above 40 Hz (Fig. 2c). In order to determine the mechanism underlying this effect, we measured the membrane potential of muscle fibres within the intact soleus muscle using sharp electrodes. We found that the action potential duration was strongly dependent on the duration of light pulses (Fig. 3a), and repolarization was delayed after termination of illumination (Fig. 3b). This can be explained by the slow off-kinetics (time constant of deactivation ∼20 ms) of ChR2(H134R)^7^. Thus, at higher repetition rates, repolarization did not occur leading to continuous depolarization (Fig. 3c).

**Figure 4:**
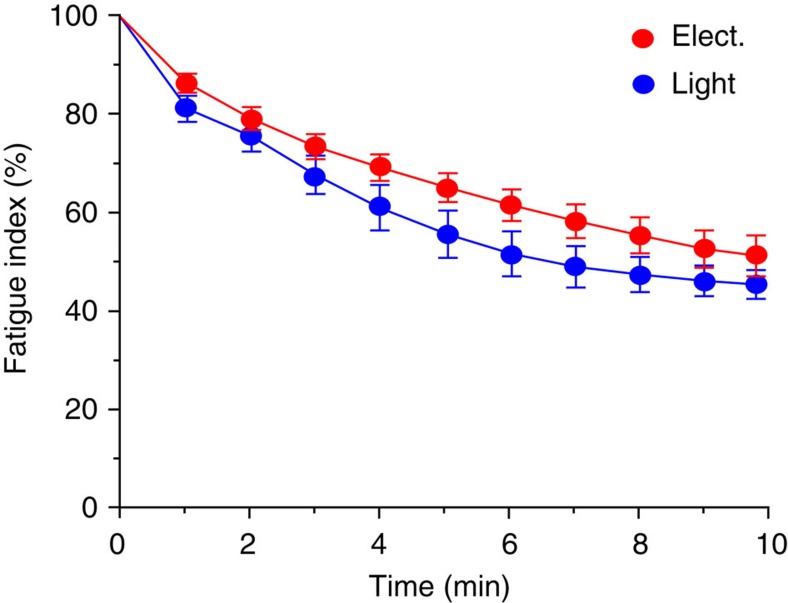
Fatigue induction during optical and electrical stimulation. Fatigue development during 350-ms-long tetanic stimulation pattern (optical, 5 ms pulses, 30 Hz, 1.4 mW mm^−2^, electrical, 0.1 ms, biphasic, 100 Hz, 20 V) over a time period of 10 min. Fatigue values were compared at each time point using a two-way, paired Student's *t*-test resulting in *P* values>0.05 (*n*=7).

**Figure 5:**
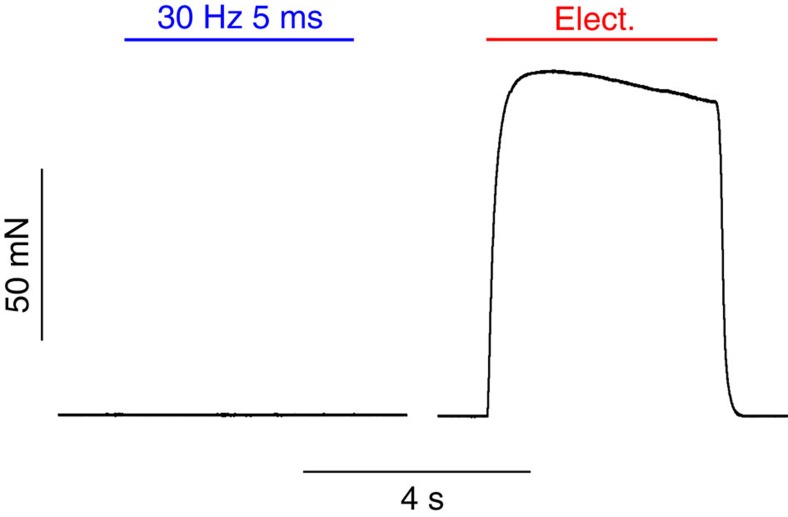
Optical stimulation in CAG EGFP control mice. Representative recording (*n*=4) of isometric force measurements of soleus muscles from mice expressing only EGFP during optical stimulation with 5-ms-long pulses at 30 Hz (1.4 mW mm^−2^) and electrical stimulation (10 mA, 1 ms, 100 Hz).

Muscle fatigue was not different between optical (5 ms, 30 Hz, 1.4 mW mm^−2^) and electrical stimulation (20 V, 0.1 ms, biphasic, 100 Hz; Fig. 4). Importantly, isometric force measurements with soleus muscles from control mice expressing EGFP did not reveal any light-induced contractions (Fig. 5) confirming the specificity of optogenetic stimulation.

### Optogenetic stimulation of explanted larynges

**Figure 6:**
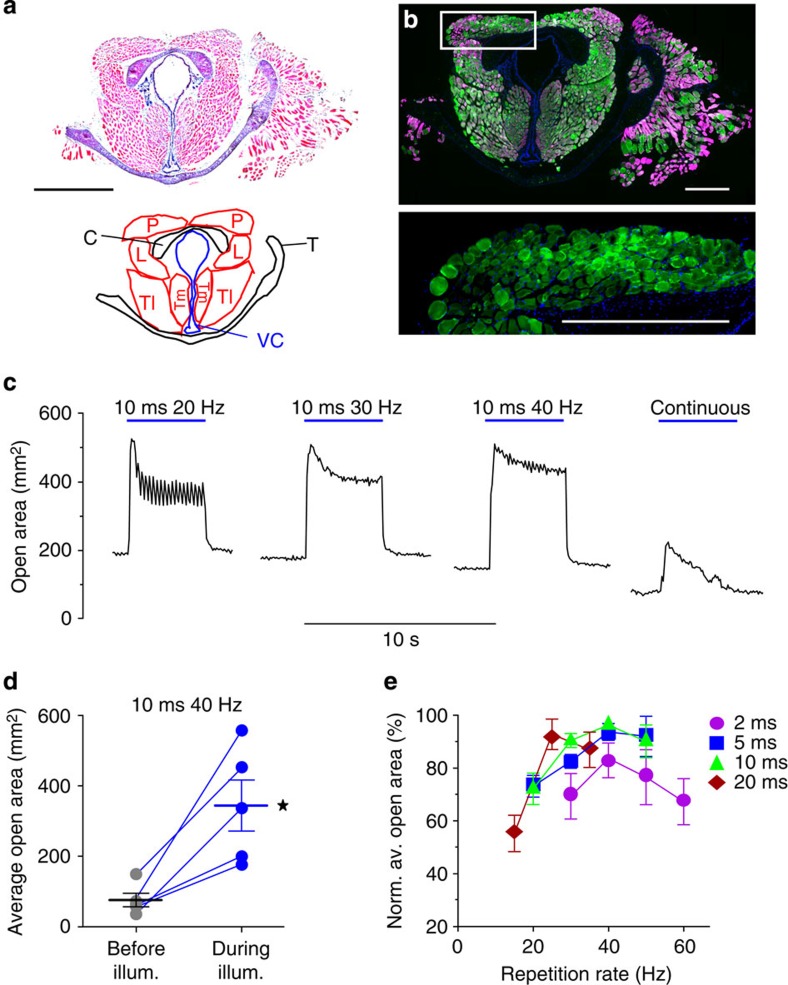
Optogenetic opening of the vocal cords. (**a**) H&E staining of a section through the larynx (top) and schematic drawing displaying the different muscle groups and cartilages (bottom: P: posterior cricoarytenoid muscle; L, lateral cricoarytenoid muscle, C, cricoid cartilage; VC, vocal cords; T, thyroid cartilage; Tl, lateral cricothyroid muscle; Tm, medial cricothyroid muscle). (**b**) Membrane-bound ChR2-EYFP signals (green, enlargement in lower panel) overlaid with α-actinin staining (magenta) in a consecutive slice. (**c**) Representative examples of opening of the vocal cords induced by various 4-s-long illumination patterns. (**d**) Open area between the vocal cords calculated before and during illumination (10 ms light pulses, 35.7 mW mm^−2^, 40 Hz repetition rate, *n*=5, two-way, paired Student's *t*-test, *P*=0.0145). (**e**) Relationship between repetition rate and average open area normalized to maximal opening for different light pulse durations (35.7 mW mm^−2^, *n*=3). Error bars, s.e.m., nuclear staining in blue, scale bars, 1 mm (**a**), 500 μm (**b**).

One clear advantage of the optogenetic stimulation approach could be the selective activation of individual agonistic and antagonistic muscles located in close vicinity. In contrast, electrical stimulation lacks this spatial precision and indirect stimulation through motoric nerves is also impossible in case that both muscle groups are innervated by the same nerve. The mammalian larynx is a good example because the recurrent laryngeal nerve innervates all skeletal muscles that control the position of the vocal cords (Fig. 6a). Contraction of the posterior cricoarytenoid muscle opens the vocal cords allowing air passage into the lung during breathing, whereas the other intralaryngeal muscles close the vocal cords for phonation and protection from aspiration^12^,^13^. We have taken advantage of optogenetic stimulation to demonstrate selective stimulation of individual intralaryngeal muscles. For this purpose, an *ex vivo* preparation of the larynx was used that reflects the physiological characteristics of a denervated larynx *in vivo* including paramedian position and physiological tension of the vocal cords as well as integrity and intact biomechanical movement of the intralaryngeal muscles and the arytenoid cartilage. In explanted larynges from the ChR2-transgenic mouse line, all muscles displayed bright and membrane-bound EYFP signals (Fig. 6b). Selective illumination of the posterior cricoarytenoid muscle was performed with a light-emitting diode (LED)-coupled small light guide (400-μm core diameter), and the most efficient stimulation pattern was determined using 4-s-long stimulations with a broad range of pulse durations (2–20 ms) and repetition rates (10–50 Hz). Illumination with low repetition rates (<20 Hz) showed incomplete tetanic contractions with oscillating movements (Fig. 6c left), whereas rates above 30 Hz led to sustained opening of the vocal cords (Fig. 6c middle, Supplementary Movie 2). Importantly, during continuous illumination we observed an initial opening with subsequent re-closure (Fig. 6c right) highlighting the importance of pulsed illumination. For quantification of vocal cord opening, we calculated the average of the area between the vocal cords at baseline and during illumination. Comparison of all tested stimulation patterns revealed that 10-ms-long light pulses at 40 Hz induced the maximum opening of the vocal cords leading to a change of the open area from 76±19 to 344±73 mm^2^, which is an increase of 420±131% (*n*=5; Fig. 6d). In order to take anatomical variations into account and to compare the effectiveness of different stimulation patterns, we normalized the respective openings to the maximal opening (Fig. 6e). Compared with the maximal opening obtained with 10-ms pulses at 40 Hz, we found that 12 other stimulation patterns had similar efficiency (*n*≥3, *P*>0.05, repeated analysis of variance (ANOVA) test with Bonferroni multiple comparison post test). Among these, 2-ms-long pulses at 40 Hz had the lowest duty cycle (8%) and its use would therefore avoid possible photo damage. To further prove selectivity of optical stimulation, we moved the light guide in front of the superior cricoarytenoid muscle, which is responsible for closure of the vocal cords in mice. Illumination of this region led to a closing of the vocal cords (Supplementary Movie 3).

### Gene transfer for optogenetic stimulation of wild-type mice

**Figure 7:**
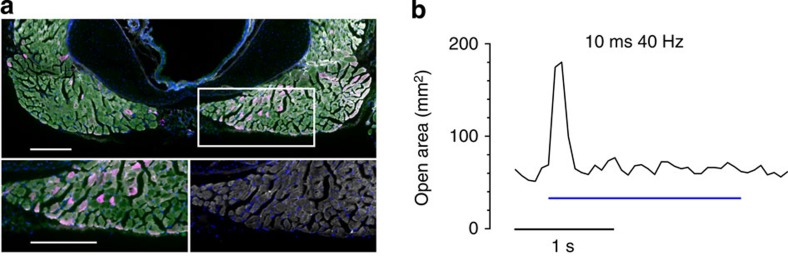
Functional ChR2 expression in the larynx after AAV-based gene transfer. (**a**) mCherry expression (magenta) in α-actinin (white)-positive fibres of the posterior cricoarytenoid muscle (autofluorescence in green, enlargements in lower panels). (**b**) Representative example of the transient opening of the vocal cords during 2-s-long pulsed illumination (10 ms, 40 Hz, 35.7 mW mm^−2^). Nuclear staining in blue, scale bar, 200 μm.

These proof-of-principle experiments were performed in a transgenic animal model, whereas for therapeutic purposes gene transfer of ChR2 into skeletal muscle is required. In order to demonstrate the feasibility of this approach for optogenetic control of larynx function in wild-type mice, we have systemically injected adeno-associated viruses (AAV) expressing ChR2(H134R) in fusion to mCherry with the capsid of serotype 9 because this serotype has been reported to infect skeletal muscles in mice^14^. Four weeks after the injection, we could detect mCherry fluorescence in the posterior cricoarytenoid muscle (Fig. 7a) and detailed histological analysis revealed that in this muscle 10.2±3.6% of the fibres (*n*=3) expressed the ChR2-mCherry fusion protein. Pulsed illumination (10 ms, 40 Hz) applied to the posterior cricoarytenoid muscle of explanted larynges from the AAV-treated mice led to an opening of the vocal cords (Fig. 7b and Supplementary Movie 4) with a change of the open area from 96±14 mm^2^ to a maximum of 227±80 mm^2^, which is an increase of 139±66% (*n*=4). The opening was only transient and declined nearly to baseline during stimulation (Fig. 7b), indicating that AAV-based ChR2 expression must be improved for optogenetic induction of sustained tetanic contractions.

## Discussion

We herein report direct optogenetic stimulation of intact mammalian skeletal muscle. Even though in skeletal muscle all of the electrically isolated fibres must be simultaneously stimulated for maximal force generation, optogenetic stimulation was found to induce 84% of the maximal force during electrical stimulation. In contrast to using brief electrical pulses, the ability to apply longer light pulses allowed the biophysical investigation of different depolarization patterns on skeletal muscle contraction, and the high spatial control enabled the selective stimulation of intralaryngeal muscles.

Using single light pulses of various duration and intensities we found increasing twitch forces either by applying higher light intensities or longer pulse durations. This can be explained with enhanced recruitment of muscle fibres from deeper layers by using higher intensities and by longer lasting ChR2 photocurrents both leading to action potential generation in more fibres. Interestingly, light pulses longer than 50 ms had no additional benefit, indicating that at this pulse duration all fibres are activated and excitation of the whole muscle is saturated. For higher and sustained forces, pulsed illumination was required because continuous illumination induced only a transient contraction, which highlights the importance of repolarization for proper tetanus induction in skeletal muscle. At low frequencies (<30 Hz), optogenetic stimulation was superior to electrical stimulation because it allowed longer pulse durations and hence the prolonged action potentials would result in longer Ca^2+^ transients and higher force generation. In contrast, at high repetition rates (>40 Hz), the slow off-kinetics of the ChR2(H134R) variant prevented the repolarization between pulses. This causes similar to constant illumination, refractoriness of L-type Ca^2+^ channels, impaired Ca^2+^ accumulation and attenuation of force^15^. Accordingly, short phases of repolarization are required for sustained tetanic contractions. Thus, in contrast to cardiac muscle, where this effect is not present because of the long action potentials and the inability to generate tetanus, larger contractile force in skeletal muscles could be generated by high-rate tetanus induction using ultrafast ChR2 variants with fast off-kinetics (time constant of deactivation ∼5 ms) such as ChETA^16^ or Chronos^17^. Furthermore, light attenuation by blood and muscle tissue could be decreased using ChR2 variants with red-shifted excitation spectra^18^ because of higher light penetrance.

Our experiments also provide proof-of-concept for a translational approach. Bilateral recurrent nerve paralysis is a severe complication of various disease processes^19^ and results in a fixed paramedian position of the vocal cords and life-threatening dyspnoea^20^. Current treatment options are surgical removal of one vocal cord or permanent tracheotomy; however, these treatment forms have severe side effects^21^,^22^. Alternatively, also electrical stimulation has been tested; however, this approach is seriously affected by corrosion or encapsulation of the electrode tip, discomfort due to the stimulation of sensory nerves and co-stimulation of antagonist muscles^20^,^23^, preventing its clinical use. Our experiments prove the specific stimulation of small intralaryngeal muscles to selectively open and close the vocal cords. Given the anatomic and biomechanical similarities between larynges from mice and humans^24^, this approach could be a potential therapeutic option for patients suffering from bilateral laryngeal paralysis.

AAVs would be a very promising tool for the expression of ChR2 because several clinical trials have already proven their efficacy and safety for therapeutic gene transfer into skeletal muscles in humans^25^,^26^,^27^. The transduction rate in the larynx could be enhanced by either using catheter-based intravascular injection into laryngeal vessels, or local intramuscular injection of AAV. Selective expression of ChR2 in skeletal muscle cells would enable pain-free optogenetic stimulation, and selective targeting of ChR2 to the most fatigue-resistant slow twitch muscle fibres could be used to reduce muscle fatigue. Moreover, direct optogenetic stimulation of muscle could be also applied in diseases that affect peripheral nerves or synaptic transmission, such as amyotrophic lateral sclerosis or myasthenia gravis in which the previously reported indirect optogenetic stimulation through peripheral nerves^4^,^5^,^6^ would fail.

In summary, we demonstrate the feasibility and the biophysical basics of direct optogenetic stimulation in intact mammalian skeletal muscle. Its high efficacy as well as the advantage of site-specific and time-controlled excitation of skeletal muscle makes this an ideal approach to explore basic principles in muscle physiology and to develop this further towards translation.

## Methods

### Transgenic mouse model

Experiments were performed using the previously reported^10^ transgenic mouse line expressing the ChR2 (H134R) variant under the control of the chicken-β-actin (CAG) promoter; in total, eight male mice (ages 2–8 months, median 2.5 months) and six females (ages 5–8 months, median 4.8 months) were used. Transgenic mice (three female mice, 7 months old) expressing EGFP under control of the CAG promoter^10^ served as controls in Fig. 5. Both lines were backcrossed ≥10 generations on a CD1 genetic background. Animal experiments conformed with the Guide for the Care and Use of Laboratory Animals published by the US National Institutes of Health (eighth edition, revised 2011). For the harvesting of tissue, mice were killed by cervical dislocation and dissection of skeletal muscle was performed in ice-cold bicarbonate Tyrode solution containing (in mM) 118 NaCl, 3.4 KCl, 0.8 MgSO_4_, 1.2 KH_2_PO_4_, 11.1 Glucose, 25 NaHCO_3_ and 2.5 CaCl_2_ gassed with 95% O_2_ and 5% CO_2_ at room temperature. Preparation of FDB muscles was performed in ice-cold Tyrode solution containing (in mM) 140 NaCl, 5.4 KCl, 1.8 CaCl_2_, 2 MgCl_2_, 10 glucose and 10 Hepes (pH 7.4, adjusted with NaOH) and preparation of explanted larynges was performed in ice-cold DPBS (Gibco, Life technologies).

### Single FDB fibre preparation

To obtain single FDB fibres, FDB muscles were explanted and dissociated with 2 mg ml^−1^ collagenase B (Roche) in Tyrode solution for 1 h at 37 °C. Single FDB fibres were plated on glass coverslips coated with 100 μg ml^−1^ Laminin (Sigma-Aldrich) and incubated for 2–3 h at room temperature in Tyrode solution. Illumination (470 nm) was performed on an Axiovert 200 microscope (Zeiss) through a × 20 Fluar objective (numerical aperture: 0.75, Zeiss) with a temperature-controlled LED module (Omicron, LEDMOD LAB 470 nm, Omicron Laserage). Videos were recorded at 60 fps with a CMOS camera (Basler A602f) and the Fire-I Application 5.51 software (UniBrain).

### Isometric force measurements

Soleus muscles were explanted and incubated in Bicarbonate Tyrode solution containing (in mM) 118 NaCl, 3.4 KCl, 0.8 MgSO_4_, 1.2 KH_2_PO_4_, 11.1 Glucose, 25 NaHCO_3_ and 2.5 CaCl_2_ gassed with 95% O_2_ and 5% CO_2_ at room temperature. To measure isometric force, soleus muscles were mounted between a force transducer (KG 2, Scientific Instruments) and a fixed hook. For illumination, a temperature-controlled LED module (Omicron, LEDMOD LAB 470 nm, Omicron Laserage) was coupled to a plastic optical fibre (2 mm diameter, numerical aperture (NA)=0.5) and directed into the window of the muscle recording chamber resulting in the illumination of the whole muscle. Light intensity was calibrated by measuring the power (PM100 powermeter with S130A sensor, Thorlabs) at the site of muscle placement within the recording chamber.

Control of LED and recording of force were performed with a Powerlab 8/30 recording system and the Chart 7.1 software (AD Instruments). Electrical stimuli were generated by a stimulator (Stim7, Scientific Instruments or stimulator 2100, AM Systems) connected to the force transducer and the hook. Soleus muscles were pre-stretched until single light stimuli (10 ms, 1.4 mW mm^−2^) evoked maximal force generation. This optimal muscle length was multiplied by 0.85 to calculate the optimal fibre length as previously reported^28^. To confirm the viability of the skeletal muscle before the optical activation, soleus muscles were activated with a maximal electrical tetanus stimulation (20 V, 0.1 ms biphasic, 100 Hz, 2 s) and, only if this muscle yielded a maximum force generation above 70 mN, it was used for further experiments. Light-induced single twitches were evoked with at least 10-s intervals between stimulations. Tetanic contractions induced by optical and electrical stimulation were 2 s long with an interval of 3 min between stimulations. Directly after force recording, tendons were removed and soleus muscles were blotted on tissue paper to remove the excess fluid and weighed. For quantification, maximal force was analysed for single twitch contractions and the average force during tetanic contractions was normalized to cross-sectional area that was calculated as weight/(optimal fibre length × density (1.06 mg mm^−3^) as previously reported^28^. For the analysis of the force–frequency relationship (Fig. 2c), force was normalized to the maximal force induced by electrical stimulation (20 V, 1 ms, biphasic) to account for variation between muscles. To analyse fatigue induction, 350-ms-long tetanic optical or electrical stimulation pattern were applied every 3.7 s over a total period of 10 min similarly as reported before using the best optical (5 ms, 30 Hz, 1.4 mW mm^−2^) and electrical (100 Hz, 20 V, 0.1 ms, biphasic) stimulation pattern^29^.

### Recording of membrane potential from intact skeletal muscles

For intracellular recording of membrane potential, soleus muscles were mounted, overstretched to 110–130% of optimal length and 25 mM 2,3-Butanedione monoxime (Sigma) was added to the bicarbonate Tyrode solution in order to alleviate contractions. Sharp electrodes (filled with 3 M KCl, 50–150 MΩ) were impaled into the soleus muscle using a piezomanipulator (PM10, Maerzhaeuser Wetzlar) and membrane potential was recorded with a bridge amplifier (BA-03X, npi electronic), the Powerlab 8/30 recording system and the Chart 7.1 software (AD Instruments). Only cells with a resting membrane potential more negative than −50 mV and action potentials with overshoot above 0 mV were included into the analysis. Action potential duration was determined by measuring the time from the peak to repolarization to −35 mV.

### Analysis of laryngeal function *ex vivo*

Explanted larynges were placed in bicarbonate Tyrode solution (for composition see above) gassed with 95% O_2_ and 5% CO_2_. For optical stimulation a light guide (Ø 400 μm, NA 0.48) was coupled to a 470-nm LED (M470F1, Thorlabs), controlled by a Powerlab 8/30 recording system and the Chart 7.1 software (AD Instruments) and placed directly in front of the posterior cricoarytenoid muscles or the superior cricoarytenoid muscles. Light intensity was calibrated at the tip of the light guide, and this might be therefore an overestimate. For visualization explanted larynges were illuminated from the top with white light from a cold-light source (Olympus KL2500LCD) and videos were recorded through a macroscope (MVX10, Olympus) equipped with a × 1 Objective (MVPLAPO1x, NA 0.25, Olympus) and a 542±27-nm bandpass filter (F37-542, AHF Analysetechnik) to block the stimulation light. For each stimulation pattern, a single video file was recorded at 15 or 30 f.p.s. with a CMOS camera (Basler A602f) and the Fire-I Application 5.51 software (UniBrain). Videos of the same larynx were merged, and only videos in which the whole area between the vocal cords was darker than the surrounding tissue of the larynges could be used for the automatic analysis. Brightness adjustments and converting into binary images by threshold analysis was performed with the Virtual Dub video processing software. This conversion resulted in a video in which the area between the vocal cords was black and the surrounding tissue turned white. A custom-made, Labview-based area analysis software detected and calculated the black area between the vocal cords over time. Calibration of pixel size was used to determine the absolute areas (in mm^2^). Traces of the open area between the vocal cords over time were further analysed using the Chart 7.1 software (AD Instruments). The average open area was analysed by determining the integral of the open area and was normalized to the maximal average opening of each muscle in order to take anatomical variations into account.

### AAV injection and analysis of efficacy

Injection of AAV was approved by the local ethics review board. The AAV9-CAG-hChR2(H134R)-mCherry virus consisting of the AAV9 virus capsid and AAV2 virus DNA were ordered from the Penn Vector Core from the University of Pennsylvania. Ten-week-old female mice (CD1, Charles River) were anaesthetized by inhalation of 1.5% isoflurane and O_2_/N_2_O (50%/50%). Overall, 2 × 10^11^ genome copies of AAV diluted in 100 μl PBS were injected slowly (∼10 s) through a 1-ml syringe (Insuline Syringe U-40, BD) connected to a sterile 33-gauge needle (200 μm diameter, Nanopass 33, Terumo) into the left jugular vein. Bleeding was carefully stopped using surgical swabs trapped in forceps, and the lesion was closed with a suture (Prolene C-1 13 mm 3/8c, Ethicon). Four weeks later, larynges were explanted and tested with the same set-up as described above using 2 or 4 s long, pulsed stimulation with a pulse duration of 10 ms and a repetition rate of 40 Hz, and the open area between the vocal cords was manually determined in individual frames using the area measurement tool of the ImageJ software (NIH).

### Histology and immunofluorescence staining

Single FDB fibres, soleus muscles and explanted larynges were fixated with 4% paraformaldehyde (PFA). Cryopreserved muscles and larynges were sectioned with a cryotome (Cryostat CM3050 S, Leica) into 10-μm-thick slices. Slices of larynges were stained with haematoxylin and eosin (H&E) according to the standard protocols and consecutive slices were used for immunohistochemical stainings. All sections were permeabilized with 0.2% Triton X in PBS, and immunohistochemical stainings were performed in 5% donkey serum for 2 h at room temperature with a primary antibody to the α-actinin protein (1:400, Sigma-Aldrich). Cy3- or Cy5-conjugated secondary antibodies (1:400, Jackson ImmunoResearch) were diluted in Hoechst 33342 for nuclear staining. Pictures of native soleus muscles and laryngeal H&E sections were acquired with a stereomicroscope (AxioZoom.V16, Zeiss), and fluorescence pictures of laryngeal slices, soleus muscles and single FDB fibre were acquired with an inverted fluorescence microscope (Axio Imager, Zeiss). To analyse the percentage of muscle fibres with ChR2-mCherry expression after systemic AAV injection, fluorescence images were acquired with a mCherry filter set (F46-008, AHF Analysentechnik) and overlaid with native autofluorescence signals acquired with a Cy2 filter set and α-actinin staining using the Cy3 channel. The number of mCherry-positive muscle fibres was counted and normalized to all muscle fibres identified by α-actinin staining.

### Statistics

Statistical data are shown as mean±s.e.m. For comparison of different stimulation patterns, repeated ANOVA tests were performed using Dunnett's multiple comparison tests for isometric force measurements with continuous light stimulation as control (Fig. 2b) or Bonferroni multiple comparison test for opening of the vocal cords. For comparison of the average open area between vocal cords before and during illumination (Fig. 6d) a one-way, paired Student's *t*-test and for comparison of fatigue induction by optical and electrical stimulation (Fig. 4) a two-way paired Student's *t*-test was chosen. The *n* values in the legends indicate the number of independent experiments (muscles or larynges), and a *P*<0.05 was considered statistically significant.

## Additional information

**How to cite this article:** Bruegmann, T. *et al*. Optogenetic control of contractile function in skeletal muscle. *Nat. Commun*. 6:7153 doi: 10.1038/ncomms8153 (2015).

## Supplementary Material

Supplementary Movie 1 Video of an isolated flexor digitorum brevis fiber

Supplementary Movie 2 Video of an explanted larynx from a transgenic mouse expressing ChR2

Supplementary Movie 3 Video showing selective stimulation of the superior cricoarytenoid muscle of an explanted larynx from a transgenic mouse expressing ChR2

Supplementary Movie 4 Video of an explanted larynx from a mouse 4 weeks after AAV-based gene transfer of ChR2- mCherry
